# Contemporary Concise Review 2025: Asthma

**DOI:** 10.1002/resp.70270

**Published:** 2026-07-01

**Authors:** Jun Miyata, Koichi Fukunaga

**Affiliations:** ^1^ Division of Pulmonary Medicine, Department of Medicine Keio University School of Medicine Tokyo Japan

**Keywords:** airway epithelium, asthma, disease modification, microbiome, oral corticosteroid, remission, treatable traits

## Abstract

- Asthma research in 2025 further advanced a multidimensional view of asthma, integrating disease trajectory, exacerbation risk, structural airway changes, comorbidities, and treatment responsiveness.
- In mild asthma, studies published in 2025 further reinforced the importance of anti‐inflammatory reliever strategies over SABA‐only treatment by supporting timely inhaled corticosteroid delivery at symptom worsening to reduce exacerbation risk, particularly in adults and adolescents, while providing emerging but still nuanced evidence in child.
- In severe asthma, the therapeutic focus expanded beyond exacerbation reduction towards disease modification, including oral corticosteroid sparing, improvement or normalisation of lung function, reduction of mucus plugging, and attainment of clinical remission.
- Clinical remission became more clearly defined and increasingly positioned as a treatment target and research endpoint, supported by emerging consensus definitions, real‐world data, and analyses of biologic trials.
- Real‐world asthma management increasingly emphasised implementation, including treatable‐traits‐based care, biomarker‐informed stratification, digital inhaler technologies, and approaches to persistent ethnic, social, and age‐related inequities in outcomes.
- Advances in pathobiology highlighted the central role of the airway epithelium, inflammatory cellular ecosystems, microbiome‐associated endotypes, mucus‐plug biology, and early‐life origins of airway remodelling in shaping asthma heterogeneity and progression.

## 1 Introduction

Asthma research has continued to evolve, while clinical practice has changed substantially in recent years, prompting a reappraisal of current management strategies. Advances in pathobiology have established asthma as a multidimensional disorder shaped by disease trajectory, exacerbation risk, structural airway changes, comorbidities, and variable treatment responsiveness. In 2025, major clinical trials, real‐world studies, consensus reports, and mechanistic investigations further advanced key themes in asthma care, including anti‐inflammatory reliever strategies in mild asthma, disease modification and oral corticosteroid (OCS) sparing in severe asthma, remission as a treatment target, and personalised management based on treatable traits and digital technologies. This review summarises the key asthma studies reported in 2025 and discusses their implications for future research and clinical practice. The major findings are summarised in Table 1.

**TABLE 1:** Key developments in asthma research in 2025.

Category	Content	References
Mild asthma	Anti‐inflammatory reliever strategies were further reinforced, with strong evidence in adults and adolescents and emerging but age‐dependent evidence in children, supporting timely inhaled corticosteroid delivery at symptom worsening rather than SABA‐only treatment.	[1, 2, 3]
Severe asthma: disease modification and OCS reduction	The therapeutic focus expanded beyond exacerbation reduction towards disease modification, including oral corticosteroid reduction, preservation or normalisation of lung function, and reduction of mucus burden.	[4, 5, 6, 7, 8, 9]
Remission and next endpoints	Clinical remission became more clearly defined as a treatment target and research endpoint, with emphasis on composite outcome measures incorporating symptoms, exacerbations, oral corticosteroid use, lung function, and patient‐centred domains.	[8, 10, 11, 12, 13, 14, 15]
Real‐world care: treatable traits and biomarker‐guided care	Real‐world asthma management increasingly emphasised implementation, including the feasibility of treatable‐traits approaches in specialist respiratory clinics and more integrated biomarker‐based stratification.	[16, 17, 18]
Real‐world care: digital implementation and health inequities	Digital inhaler technologies showed potential to improve long‐term asthma control and support new trial designs, while persistent ethnic and community‐level inequities remained important determinants of outcome.	[19, 20, 21, 22, 23]
Pathobiology: epithelium and immune ecosystems	Advances in epithelial biology and spatial immunology highlighted rare epithelial‐cell plasticity, epithelial danger sensing, and pro‐inflammatory cellular ecosystems as key determinants of asthma heterogeneity.	[24, 25, 26, 27, 28]
Pathobiology: mucus plugging and exacerbation	Mucus plugs were further characterised as structural lesions maintained by epithelial remodelling and inflammatory‐cell interactions, while impaired antiviral epithelial responses were linked to exacerbation susceptibility.	[29, 30, 31]
Pathobiology: microbiome, ACO, and eosinophil biology	Microbiome‐associated endotypes, early‐life ACO models, and eosinophil‐focused multi‐omics and single‐cell studies further expanded understanding of structural disease and immune heterogeneity.	[32, 33, 34, 35, 36]

Abbreviations: ACO, asthma–COPD overlap; OCS, oral corticosteroid; SABA, short‐acting β2‐agonist.

## 2 Mild Asthma

**Summary**

- Mild asthma should not be regarded as benign, as severe exacerbations may occur despite relatively mild daily symptoms.
- Anti‐inflammatory reliever strategies were more effective than short‐acting β2‐agonist (SABA)‐only treatment in adults and adolescents, with emerging evidence in children that requires age‐specific interpretation.
- Management of mild asthma is shifting from symptom relief alone towards timely anti‐inflammatory treatment to reduce future risk.

Anti‐inflammatory reliever (AIR) therapy refers to the use of an inhaled corticosteroid (ICS)‐containing reliever, such as ICS–formoterol or ICS–SABA, at the time of symptom worsening. The evidence base supporting AIR therapy in mild asthma was already established before 2025, particularly in adults and adolescents, through trials of as‐needed budesonide–formoterol, including SYGMA, Novel START, and PRACTICAL, and by subsequent evidence syntheses of ICS–formoterol reliever strategies [37, 38, 39, 40, 41]. In 2025, new evidence further reinforced the superiority of ICS‐containing reliever strategies over SABA‐only treatment, while extending discussion to additional regimens and age groups. Mild asthma should not be regarded as benign, because even patients with mild daily symptoms remain at risk of severe exacerbations, and treatment directed solely at symptom relief is insufficient. The key message from studies published in 2025 was therefore not that the AIR concept was newly established, but that timely delivery of anti‐inflammatory treatment at symptom worsening remains central to reducing future exacerbation risk (Table 2) [1, 2, 3].

**TABLE 2:** Major clinical trials cited in this review of asthma published in 2025.

Disease	Trial	Drug/intervention	Study population	Key findings	References
Mild asthma	BATURA	As‐needed albuterol–budesonide	Patients aged ≥ 12 years with inadequately controlled mild asthma	Reduced the risk of severe exacerbations and lowered systemic glucocorticoid exposure compared with as‐needed albuterol alone; safety was similar between groups	[1]
Mild asthma	CARE	As‐needed budesonide–formoterol	Children aged 5–15 years with mild asthma	Reduced asthma attacks and prolonged the time to first attack in the overall 5–15‐year cohort compared with as‐needed salbutamol; subgroup findings indicated greater uncertainty in younger children; safety was broadly similar.	[2]
Severe uncontrolled asthma	WAYFINDER	Tezepelumab	Adults with corticosteroid‐dependent, severe uncontrolled asthma	In a single‐arm phase 3b study, enabled substantial reduction in maintenance OCS use, and approximately half of patients discontinued OCS completely.	[4]
Moderate‐to‐severe asthma	VESTIGE	Dupilumab	Patients with moderate‐to‐severe asthma	Reduced mucus plug burden and improved lung function, particularly in patients with high baseline mucus plug scores	[7]

Abbreviation: OCS, oral corticosteroid.

Within this established framework, BATURA, published in the *New England Journal of Medicine* in 2025, provided important confirmatory and extending evidence in adolescents and adults. This fully virtual, decentralised, phase 3b trial compared as‐needed fixed‐dose albuterol–budesonide with as‐needed albuterol alone in patients aged 12 years or older with inadequately controlled mild asthma [1]. As‐needed albuterol–budesonide significantly reduced the risk of severe exacerbations and lowered systemic glucocorticoid exposure, with a similar adverse‐event profile. These findings confirmed and extended previous evidence from as‐needed budesonide–formoterol studies by showing that an ICS–SABA reliever strategy also reduces exacerbation risk compared with SABA alone.

In children, the CARE trial, published in The *Lancet*, provided important but more nuanced evidence [2]. In this 52‐week, open‐label, multicentre, randomised trial of children aged 5–15 years with mild asthma, as‐needed budesonide–formoterol reduced asthma attacks and prolonged the time to first attack compared with as‐needed salbutamol, with broadly similar safety. However, prespecified subgroup analyses suggested a larger treatment effect in older children aged 12–15 years than in younger children aged 5–11 years. The authors discussed several possible explanations, including delayed reliever use in younger children requiring caregiver recognition or assistance and less frequent eosinophilic‐driven asthma attacks in younger children. Thus, CARE provided direct paediatric evidence for a concept previously established in adults and adolescents, while also highlighting the need for further age‐specific evaluation in younger children.

The broader significance of this treatment principle was reinforced by a systematic review and meta‐analysis published in *JAMA*, which quantitatively integrated the pre‐existing and recent evidence [3]. Across 27 randomised trials, both ICS–SABA and ICS–formoterol reliever strategies reduced severe exacerbation risk compared with SABA alone, without increasing serious adverse events. However, the evidence base was not uniform across age groups, and relatively few trials included children younger than 12 years. Therefore, the conclusion that ICS‐containing reliever strategies reduce exacerbation risk is strongest for adults and adolescents, whereas the role of AIR therapy in younger children should be interpreted with caution and requires further age‐specific evaluation. Management of mild asthma is therefore moving away from SABA‐only treatment towards a more preventive, risk‐based approach, particularly in populations for which the evidence base is mature. Collectively, these findings suggest that mild asthma should be viewed as an early and modifiable stage of disease.

## 3 Disease Modification and OCS Reduction in Severe Asthma

**Summary**

- The management of severe asthma is shifting beyond exacerbation control alone towards disease modification and remission.
- Biologic therapies may reduce oral corticosteroid dependence, improve lung function, and reduce mucus burden in selected patients.
- Residual disease processes, including mucus‐related pathology and airway infection, remain important barriers to remission in severe asthma.

In 2025, the goals of severe asthma management expanded beyond exacerbation prevention towards disease modification, including withdrawal from OCS dependence, stabilisation or normalisation of lung function, and the attainment of remission. The value of biologic therapies is therefore increasingly judged not only by their ability to reduce exacerbations, but also by their capacity to reduce systemic corticosteroid exposure, improve structural and functional abnormalities, and potentially modify long‐term disease course (Table 2) [4, 5, 6, 7, 8, 9].

This shift was illustrated by the WAYFINDER trial [4]. In adults with OCS‐dependent, severe uncontrolled asthma, tezepelumab enabled most patients to achieve substantial reductions in maintenance OCS, and approximately half discontinued OCS completely, with broadly consistent effects across biomarker‐defined subgroups. Notably, this contrasts with the earlier SOURCE trial, which did not meet its primary endpoint for OCS reduction overall, despite a signal in patients with higher baseline eosinophil counts [5]. Several factors may explain this difference, including differences in trial design, OCS‐tapering procedures, patient selection, baseline biomarker distribution, and the absence of a placebo comparator in the single‐arm WAYFINDER study. Thus, WAYFINDER supports the potential role of tezepelumab in facilitating OCS reduction or discontinuation in selected patients with OCS‐dependent severe asthma, but direct comparisons with SOURCE should be interpreted cautiously, and the extent to which this effect is independent of baseline T2 biomarker status requires further clarification.

The significance of tezepelumab, however, extends beyond OCS reduction. In a pooled post hoc analysis of the PATHWAY and NAVIGATOR studies, tezepelumab improved pre‐bronchodilator FEV_1_ and increased the proportion of patients who achieved normalisation of lung function, particularly in those with higher T2 biomarker levels and shorter disease duration [6]. These findings suggest that early introduction of effective therapy may help redirect lung function trajectories before irreversible airflow limitation becomes established.

Mucus plugging also emerged more clearly as a structural treatable trait in severe asthma. In the VESTIGE trial, dupilumab reduced mucus plug burden, with particularly marked improvement in lung function among patients with high baseline mucus plug scores [7]. This study established mucus plugging not simply as an imaging abnormality, but as a reversible therapeutic target linked to functional improvement.

These individual trials were further supported by remission research. A systematic review and meta‐analysis published in *Lancet Respiratory Medicine* showed that clinical remission is achievable in a substantial proportion of patients receiving biologic therapy, while also identifying lower lung function, greater symptom burden, longer disease duration, maintenance OCS use, obesity, and depression as barriers to remission [8]. In parallel, Nolasco and colleagues reported that a comprehensive strategy guided by airway inflammometry and bioimaging was associated with high rates of OCS‐free status and clinical remission, and that residual disease activity was explained less by persistent type 2 inflammation than by airway infection, mucus, and airway hyperresponsiveness [9]. Collectively, these findings suggest that disease modification in severe asthma should be viewed not simply as suppression of T2 inflammation by biologics, but as a broader strategy to reduce OCS dependence, preserve lung function, and address residual disease processes that may otherwise hinder progression towards remission, with clinical remission representing its clearest clinical manifestation through sustained control across multiple domains.

## 4 Defining Remission and Future Directions: Guideline Updates and the Next Endpoints

**Summary**

- Asthma remission is increasingly recognised as a clinically relevant treatment target and research endpoint in severe asthma.
- Future remission endpoints are likely to incorporate symptoms, exacerbations, oral corticosteroid use, lung function, and patient‐centred outcomes.
- Further refinement of remission definitions will require consideration of confounding factors, incorporation of the patient perspective, and support from guideline development and digital innovation.

**FIGURE 1:**
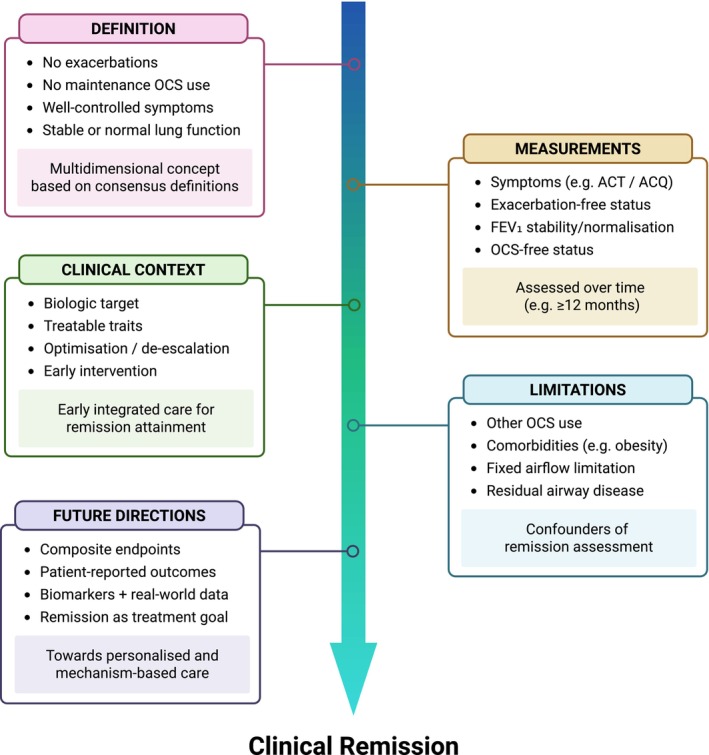
Conceptual framework of clinical remission in asthma. Clinical remission in asthma is a multidimensional and time‐dependent construct integrating symptoms, exacerbations, oral corticosteroid (OCS) use, and lung function. Core defining features include the absence of exacerbations, no requirement for maintenance OCS, well‐controlled symptoms, and stable or normalised lung function. Assessment should be based on composite endpoints evaluated over a sustained period (e.g., ≥ 12 months). In clinical practice, remission represents a treatment goal guiding biologic use, optimisation, and potential de‐escalation within a treatable‐traits framework. However, interpretation may be influenced by confounders such as comorbidities, non‐asthma‐related OCS use, and fixed airflow limitation. Future directions include standardisation of remission definitions, incorporation of patient‐reported outcomes, and integration of biomarkers and real‐world data to support personalised, mechanism‐based care.

In 2025, asthma remission became more clearly established as a practical research endpoint and therapeutic target rather than a purely aspirational goal. Thomas and colleagues emphasised remission as a multidimensional construct and highlighted the heterogeneity of current definitions and the resulting variability in reported remission rates [10]. Likewise, a consensus summary on asthma remission emphasised the need for a shared definition based on sustained clinical improvement and maintenance of control across core domains, including symptoms, exacerbations, systemic corticosteroid use, and lung function [11]. Together, these developments indicate that the therapeutic goal in severe asthma is shifting from improved control towards remission (Figure 1).

This international movement was also reflected in Japan, where a modified Delphi process and comprehensive review proposed a remission framework for the Japanese asthma prevention and management guidelines (JGL 2024), with core elements including the absence of exacerbations, good symptom control, no requirement for maintenance OCS use, and optimisation or stabilisation of lung function [12]. At the same time, a review in *JACI: In Practice* emphasised that remission should be viewed not simply as the result of biologic selection, but as the outcome of comprehensive management based on pulmonary, extrapulmonary, and behavioural treatable traits [13].

Importantly, in 2025, greater clarity emerged not only regarding the definition of remission, but also regarding its attainability and associated clinical factors. A systematic review and meta‐analysis published in *Lancet Respiratory Medicine* showed that remission can be achieved in a substantial proportion of patients with severe asthma treated with biologics, whereas lower lung function, greater symptom burden, longer disease duration, maintenance OCS use, obesity, and depression were associated with lower rates of remission attainment [8]. In this context, a biologics‐focused review on asthma remission further suggested that remission is more likely in patients with a type 2 endotype, milder disease, shorter disease duration, and fewer comorbidities [14]. Collectively, these findings support a shift beyond exacerbation reduction alone towards standardised composite endpoints that incorporate steroid‐free status, stabilisation or normalisation of lung function, and broader patient‐centred domains. At the same time, Thomas and colleagues emphasised that remission assessment should account for important confounding factors, including OCS use for other indications, obesity, fixed airflow obstruction, and inducible laryngeal obstruction, and that further refinement of remission definitions will require explicit incorporation of the patient perspective [10].

From a guideline perspective, the 2025 Global Initiative for Asthma (GINA) strategy report and its subsequent minor update reflected an ongoing shift towards more precise diagnosis, biomarker interpretation, and assessment of future risk [15]. In parallel, artificial intelligence (AI) and machine‐learning approaches are beginning to be applied to asthma, particularly for real‐world phenotyping and prediction of treatment response [42, 43, 44]. Recent studies have used machine learning applied to electronic health records to classify severe asthma phenotypes and machine‐learning‐derived genetic risk scores to explore predictors of biologic response in moderate‐to‐severe asthma [45, 46]. However, these approaches remain exploratory, and AI‐driven tools have not yet been sufficiently validated to define or determine major remission endpoints in severe asthma. Therefore, remission assessment should currently rely on standardised composite clinical endpoints, while AI may support future refinement and implementation of such endpoints.

## 5 Real‐World and Community Management: Treatable Traits, Digital Implementation, and Health Inequities

**Summary**

- Real‐world asthma care is increasingly focused on implementation, including the feasibility of treatable traits approaches in specialist respiratory clinics and the broader challenges of translating such approaches into routine practice.
- Diagnosis and treatment stratification require integrated assessment rather than reliance on single biomarkers, including blood eosinophil counts alone.
- Digital technologies and equity‐oriented strategies are becoming increasingly important for improving long‐term outcomes across diverse asthma populations.

In 2025, asthma research increasingly focused not only on therapeutic advances themselves, but also on their implementation in routine care, including which patients should receive them and in which clinical settings. In real‐world care, major themes included implementing treatable‐traits approaches within specialist respiratory clinics, avoiding overreliance on single biomarkers for diagnosis and treatment stratification, translating digital technologies into meaningful long‐term benefit, and addressing social and regional inequities as modifiable determinants of outcome [16, 17, 18, 19, 20, 21, 22, 23].

A feasibility study published in *Respirology* provided evidence for the feasibility of applying a protocolised treatable‐traits approach in specialist respiratory clinics [16]. The study showed that protocolised trait assessment was acceptable and associated with improved asthma control and reduced airflow limitation but also highlighted that targeting T2 inflammation and airflow limitation alone is insufficient in many patients. From a diagnostic perspective, a *Respirology* commentary cautioned against treating the blood eosinophil count as a diagnostic shortcut for asthma, arguing that diagnosis should remain grounded in clinical context and objective testing [17]. In contrast, the PRISM analysis showed that combined assessment of blood eosinophils and fractional exhaled nitric oxide (FeNO) improved stratification of biologic responsiveness, supporting a more integrated biomarker‐based approach rather than reliance on eosinophils alone [18].

Digital implementation also evolved in 2025, from being viewed mainly as a means of improving short‐term adherence to being recognised as a strategy for improving long‐term asthma control [19, 20, 21]. The ACCEPTANCE trial showed that an electronically monitored inhaler linked to an application improved asthma control over 12 months in primary care, despite attenuation of adherence differences over time [19]. A systematic review and meta‐analysis further showed that patient‐facing digital inhalers improve asthma control and may reduce severe exacerbations in high‐risk patients, although gains in quality of life were limited and practical barriers such as device malfunction and smartphone synchronisation remained important [20]. A *Lancet Respiratory Medicine* Personal View extended this discussion by suggesting that smart inhalers, wearables, and sensors may also reshape asthma trial design through decentralised monitoring and person‐centred endpoints [21].

Health inequities and community‐level management were also prominent themes, and papers published in *Respirology* played a central role in this area [22, 23]. A nationwide study in Aotearoa New Zealand showed that wider uptake of budesonide/formoterol was accompanied by declining asthma hospital discharges in both Māori and non‐Māori populations, yet substantial ethnic inequities persisted [22]. Similarly, an Australian whole‐of‐population cohort study showed a high burden of readmission after childhood asthma hospitalisation, particularly in younger children and in the early post‐discharge period, with substantial associated healthcare costs [23]. Together, these findings suggest that future priorities in real‐world and community asthma management should extend beyond optimising outpatient prescribing to include identification of high‐risk patients, early follow‐up after discharge, and intervention strategies tailored to age, ethnicity, and socioeconomic vulnerability.

## 6 Advances in Pathobiology: Epithelium, Immunity, and Microbiome

**Summary**

- Asthma pathobiology is increasingly understood as a multilayered process involving epithelial dysfunction, immune signalling, microbiome changes, and airway remodelling.
- Epithelial plasticity, spatial inflammatory ecosystems, mucus‐plug biology, and eosinophil heterogeneity emerged as key determinants of asthma heterogeneity.
- Microbiome‐associated endotypes and early‐life asthma–COPD overlap (ACO) models provide further insight into disease progression, exacerbation risk, and structural airway abnormalities.

**FIGURE 2:**
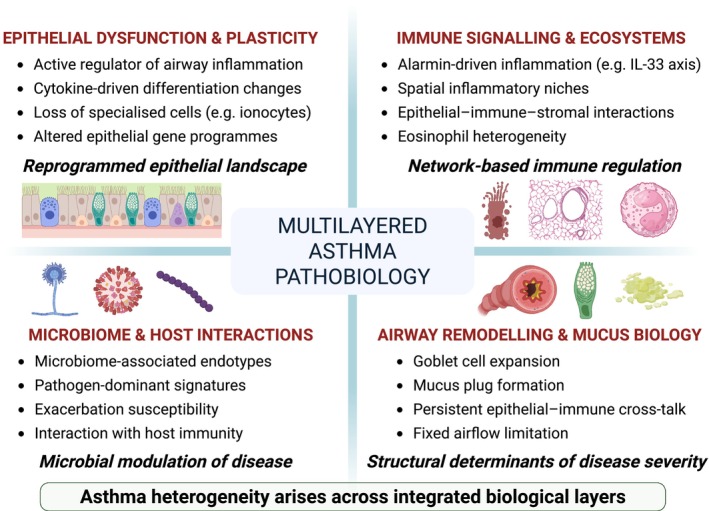
Multilayered framework of asthma pathobiology. Recent advances support a view of asthma as a multilayered disease arising from coordinated abnormalities across epithelial, immune, microbial, and structural compartments. The airway epithelium acts as an active regulator, showing altered differentiation, loss of specialised cell types, and cytokine‐driven transcriptional changes. Immune responses are organised within spatially defined cellular ecosystems involving epithelial, stromal, and immune cell interactions, including heterogeneous eosinophil programmes. Microbiome‐associated endotypes further contribute to disease heterogeneity and exacerbation risk through host–microbial interactions. Structural changes, including goblet cell expansion, mucus plugging, and airway remodelling, represent key determinants of persistent airflow limitation and disease severity. Together, these findings indicate that asthma heterogeneity is shaped by dynamic, interconnected biological layers rather than by a single inflammatory pathway.

In 2025, asthma pathobiology moved further beyond a simple inflammatory‐cell framework towards a more layered understanding of the disease, incorporating epithelial differentiation abnormalities, spatial inflammatory niches, microbiome dysfunction, eosinophil heterogeneity, and chronic remodelling originating early in life (Figure 2). The airway epithelium is increasingly viewed not merely as a passive barrier, but as a central regulatory tissue that responds to allergens, viruses, and cytokine environments and, in turn, shapes immune activation and structural airway change [24, 25, 26, 27, 28, 29, 30, 31, 32, 33, 34, 35, 36].

From the perspective of epithelial biology, rare epithelial‐cell plasticity emerged as an important theme. A *Respirology* study showed reduced numbers of CFTR‐expressing ionocytes together with impaired CFTR function in non‐eosinophilic asthma, suggesting that a neutrophilic cytokine milieu may drive ionocyte loss [24]. In parallel, a *Nature Communications* study identified tuft–ionocyte progenitor cells and showed that type 2 and type 17 cytokines can divert their differentiation away from the ionocyte lineage towards mature tuft cells [25]. Together, these findings suggest that altered epithelial‐cell composition in asthma reflects inflammatory rewiring of differentiation programmes rather than simple cell depletion.

Important advances were also made at the interface between the epithelium and the immune system. In the *Journal of Allergy and Clinical Immunology* (JACI), allergen exposure was shown to activate epithelial P2Y2 receptors, promoting ATP release, IL‐33 release, and type 2 immune responses, thereby establishing the airway epithelium as an early danger sensor [26]. Another *JACI* study identified bronchial epithelial genes associated with type 2 eosinophilic inflammation, indicating that epithelial transcriptional programmes themselves contribute to defining the inflammatory state [27]. In addition, single‐cell spatial analysis in *Nature Immunology* revealed discrete proinflammatory cellular ecosystems within the asthmatic airway wall, characterised by high alarmin and chemokine expression and close interactions among epithelial, stromal, endothelial, and mast‐cell populations [28]. These studies support a model in which asthma inflammation is sustained not only by individual cell types, but also by spatially organised cellular neighbourhoods.

The biology of mucus plugs also emerged as a major theme. In *American Journal of Respiratory and Critical Care Medicine* (*AJRCCM*), severe and fatal asthma bronchioles showed depletion of distal airway secretory cells, expansion of MUC5AC‐positive goblet cells, and formation of MUC5AC‐dominant mucus plugs, suggesting that abnormal distal epithelial differentiation underlies mucus plugging [29]. In *Journal of Clinical Investigation* (*JCI*), mucus plugs were further shown to form acutely through goblet‐cell degranulation in remodelled airways and to persist through chronic mucin–epithelium–granulocyte cross‐talk [30]. These findings indicate that mucus plugs are structural lesions maintained by epithelial remodelling and inflammatory‐cell interactions, rather than simple airway secretions.

Recent studies provided further insight into the biological basis of exacerbation susceptibility. A *JCI Insight* study in children with exacerbation‐prone asthma showed increased rhinovirus replication and exaggerated downstream interferon, inflammatory, and epithelial‐stress responses on a background of low baseline interferon‐stimulated gene expression, supporting the concept that impaired early antiviral epithelial responses contribute to exacerbation risk [31]. With regard to the microbiome, a *Respirology* study identified a multidrug‐resistant *Pseudomonas aeruginosa* ‐dominant severe asthma endotype associated with more frequent exacerbations, a resistant microbial signature, and virulence programmes related to biofilm formation and motility, independent of coexisting bronchiectasis [32].

Additional studies further expanded this framework. An early‐life‐origin ACO model showed that blockade of CD131, the common β‐chain shared by the IL‐3, IL‐5, and GM‐CSF receptors, suppressed airway hyperresponsiveness, inflammation, fibrosis, emphysema, and virus‐induced exacerbation without impairing viral clearance, supporting the concept of asthma and COPD overlap as part of a continuum of chronic inflammatory and structural airway disease [33]. Allele‐specific microRNA‐mediated regulation of ADAM33, an asthma susceptibility gene implicated in airway remodelling, in childhood allergic asthma further linked genetic background to epithelial and structural disease mechanisms [34]. Eosinophil biology also provided important new insights: all‐trans retinoic acid reversed inflammatory transcriptomic, proteomic, and lipidomic signatures in eosinophils from severe asthma, highlighting the plasticity of eosinophil activation [35], while single‐cell analysis in childhood asthma revealed distinct eosinophil‐associated airway immune programmes and functionally heterogeneous eosinophil subsets [36]. Together, these findings reinforce the view that asthma heterogeneity arises from coordinated abnormalities across epithelial, immune, microbial, and structural compartments (Figure 2).

## 7 Summary

Asthma research in 2025 further refined current management through stronger evidence supporting anti‐inflammatory reliever strategies in mild asthma, progress in disease modification and OCS reduction in severe asthma, and more clearly defined and implementable concepts of remission. Real‐world care also evolved, with increasing emphasis on treatable traits, digital technologies, and persistent health inequities. At the same time, pathobiological studies of the epithelium, immunity, the microbiome, mucus plugs, and eosinophils reinforced the view of asthma as a multilayered and dynamic disease.

Collectively, these advances indicate that asthma management is moving beyond a uniform stepwise approach towards more individualised care that incorporates future risk, biological heterogeneity, structural changes, and comorbid conditions. Future efforts should focus on integrating standardised remission criteria, implementable treatable‐traits strategies, and biologically informed interventions to enable earlier and more precise asthma care.

## Author Contributions

J.M. conceived the overall structure of the review. J.M. drafted the initial manuscript. K.F. contributed to the literature review and critical revision of the manuscript for important intellectual content; J.M. and K.F. read and approved the final manuscript.

## Funding

The authors have nothing to report.

## Conflicts of Interest

J.M. has received lecture fees from Sanofi, AstraZeneca, and GSK. K.F. has received lecture fees from Sanofi, AstraZeneca, GSK, Novartis, Boehringer Ingelheim, and Kyorin. The authors declare that they have no other competing interests related to this manuscript.

## Data Availability

Data sharing not applicable to this article as no datasets were generated or analysed during the current study.
